# Supplementary material for: Factors Influencing Workplace Health Promotion Interventions for Workers in the Semiconductor Industry According to Risk Levels of Chronic Disease
Source: Int J Environ Res Public Health. 2021 Oct 29;18(21):11383. doi: 10.3390/ijerph182111383 (PMC8583288; doi:10.3390/ijerph182111383)
Supplement: Supplementary file 1 [file ijerph-18-11383-s001.zip › ijerph-1398593-supplementary.pdf]

Table S1. Demographic characteristics of workers at risk of chronic diseases at semiconductor/liquid crystal display (LCD) workplaces by their risk classification

Characteristics		Low-risk group, n (%)		Intermediate-risk group, n (%)		High-risk group, n (%)	
total		3,961	(45.6)	3,792	(43.7)	929	(10.7)
general characteristics	sex						
	male	3,148	(42.2)	3,503	(46.9)	813	(10.9)
	female	813	(66.7)	289	(23.7)	116	(9.5)
	age groups						
	≤ 20’s	480	(59.9)	241	(30.0)	81	(10.1)
	30’s	2,140	(47.5)	1,948	(43.2)	421	(9.3)
	40’s	1,189	(40.0)	1,415	(47.6)	366	(12.3)
	≥ 50’s	152	(37.9)	188	(46.9)	61	(15.2)
work characteristics	division						
	device solution	3,367	(44.5)	3,374	(44.6)	822	(10.9)
	LCD	594	(53.1)	418	(37.4)	107	(9.6)
	work type						
	office work	1,746	(44.5)	1,784	(45.4)	398	(10.1)
	production	2,215	(46.6)	2,008	(42.2)	531	(11.2)
	industry						
	fabrication	1,706	(48.1)	1,434	(40.5)	404	(11.4)
	support	325	(46.6)	295	(42.3)	78	(11.2)
	development	1,921	(43.4)	2,056	(46.5)	447	(10.1)
	part-time	9	(56.3)	7	(43.8)	0	(0.0)
	type of work shift						
	normal working hours	3,109	(43.9)	3,215	(45.4)	758	(10.7)
	4 sets of 3 shifts	612	(58.8)	325	(31.3)	103	(9.9)
	variable shift	231	(42.5)	245	(45.0)	68	(12.5)
	flexible schedule	9	(56.3)	7	(43.8)	0	(0.0)
	physical activities at the workplace, hours						
	< 2	2,565	(45.3)	2,510	(44.3)	592	(10.4)
	2 to < 4	1,213	(44.2)	1,216	(44.3)	313	(11.4)
	4 to < 6	183	(67.0)	66	(24.2)	24	(8.8)
	workplace						
	A	864	(42.8)	917	(45.4)	238	(11.8)

Characteristics		Low-risk group, n (%)		Intermediate-risk group, n (%)		High-risk group, n (%)	
	B	1,551	(45.0)	1,513	(43.9)	382	(11.1)
	C	555	(42.4)	637	(48.6)	118	(9.0)
	D	436	(50.9)	329	(38.4)	92	(10.7)
	E	555	(52.9)	396	(37.7)	99	(9.4)
	duration employed, years						
	≤ 5	499	(50.0)	408	(40.9)	91	(9.1)
	6–10	1,108	(48.7)	941	(41.3)	227	(10.0)
	11–20	1,701	(44.7)	1,728	(45.4)	377	(9.9)
	> 20	653	(40.8)	715	(44.6)	234	(14.6)
household information	number of household members						
	1	388	(47.7)	322	(39.6)	104	(12.8)
	2	475	(46.7)	449	(44.1)	94	(9.2)
	3–5	2,829	(45.3)	2,750	(44.1)	662	(10.6)
	6–9	269	(44.2)	271	(44.5)	69	(11.3)
	household types						
	single-person household	388	(47.7)	322	(39.6)	104	(12.8)
	couple	363	(46.3)	363	(46.3)	58	(7.4)
	couple + children	1,764	(42.9)	1,913	(46.5)	436	(10.6)
	couple + parents	160	(45.7)	154	(44.0)	36	(10.3)
	couple + children + parents	519	(43.8)	539	(45.4)	128	(10.8)
	others	767	(53.4)	501	(34.9)	167	(11.6)
	dormitory						
	yes	3,850	(45.4)	3,720	(43.9)	906	(10.7)
	no	111	(53.9)	72	(35.0)	23	(11.2)
exposure to hazardous factors	special health checkups						
	not applicable	1,458	(43.0)	1,556	(45.9)	373	(11.0)
	subject of examination	2,503	(47.3)	2,236	(42.2)	556	(10.5)

Table S2. Demographic characteristics of employees at semiconductor/liquid crystal display (LCD) workplaces by risk group of cardiovascular diseases

**Characteristics**		**10-year ASCVD risk score, n (%)**				**Framingham risk score, n (%)**					
		< 7.5%		≥ 7.5%		< 10%		10 to < 20%		≥ 20%	
	total	7,352	(98.7)	95	(1.3)	23,005	(87.0)	2,965	(11.2)	486	(1.8)
general characteristics	sex										
	male	6,898	(98.7)	89	(1.3)	17,195	(83.3)	2,964	(14.4)	486	(2.4)
	female	454	(98.7)	6	(1.3)	5,810	(100.0)	1	(< 0.1)	0	NA
	age groups										
	≤ 20's	0	NA	0	NA	0	NA	0	NA	0	NA
	30's	0	NA	0	NA	17,318	(92.4)	1,220	(6.5)	195	(1.0)
	40's	6,691	(99.3)	47	(0.7)	5,294	(75.6)	1,450	(20.7)	263	(3.8)
	≥ 50's	661	(93.2)	48	(6.8)	393	(54.9)	295	(41.2)	28	(3.9)
work characteristics	Division										
	device solution	6,849	(98.8)	86	(1.2)	20,187	(86.7)	2,664	(11.4)	438	(1.9)
	LCD	503	(98.2)	9	(1.8)	2,818	(89.0)	301	(9.5)	48	(1.5)
	work type										
	office work	4,525	(98.5)	71	(0.4)	10,665	(85.4)	1,581	(12.7)	244	(2.0)
	production	2,827	(99.2)	24	(0.1)	12,340	(88.4)	1,384	(9.9)	242	(1.7)
	Industry										
	fabrication	1,722	(99.0)	18	(1.0)	9,010	(89.2)	930	(9.2)	165	(1.6)
	support	851	(97.5)	22	(2.5)	1,813	(83.4)	308	(14.2)	54	(2.5)
	development	4,740	(98.9)	54	(1.1)	12,051	(85.8)	1,727	(12.3)	267	(1.9)
	part-time	39	(97.5)	1	(2.5)	131	(100.0)	0	NA	0	NA
	type of work shift										
	normal working hours	7,139	(98.8)	90	(1.2)	18,138	(84.9)	2,791	(13.1)	440	(2.1)
	4 sets of 3 shifts	124	(97.6)	3	(2.4)	3,220	(96.4)	93	(2.8)	28	(0.8)
	variable shift	50	(98.0)	1	(2.0)	1,516	(93.9)	81	(5.0)	18	(1.1)
	flexible schedule	39	(97.5)	1	(2.5)	131	(100.0)	0	NA	0	NA
	physical activities at the workplace, hours										
	< 2	5,788	(98.7)	76	(1.3)	15,721	(86.8)	2,058	(11.4)	325	(1.8)
	2 to < 4	1,528	(98.8)	19	(1.2)	6,290	(85.6)	897	(12.2)	161	(2.2)
	4 to < 6	36	(100.0)	0	NA	994	(99.0)	10	(1.0)	0	NA
	workplace										
	A	1,839	(98.6)	27	(1.4)	5,234	(86.3)	694	(11.4)	137	(2.3)
	B	2,999	(99.0)	29	(1.0)	8,977	(86.2)	1,257	(12.1)	182	(1.7)

	Characteristics	10-year ASCVD risk score, n (%)				Framingham risk score, n (%)					
		< 7.5%		≥ 7.5%		< 10%		10 to < 20%		≥ 20%	
	C	1,547	(98.7)	20	(1.3)	3,832	(87.0)	501	(11.4)	71	(1.6)
	D	521	(97.9)	11	(2.1)	2,298	(89.0)	233	(9.0)	52	(2.0)
	E	446	(98.2)	8	(1.8)	2,664	(89.2)	280	(9.4)	44	(1.5)
	duration employed, years										
	≤ 5	274	(98.6)	4	(1.4)	3,444	(95.8)	129	(3.6)	23	(0.6)
	6–10	545	(99.3)	4	(0.7)	6,577	(93.6)	393	(5.6)	57	(0.8)
	11–20	3,385	(99.4)	20	(0.6)	10,683	(86.6)	1,429	(11.6)	228	(1.8)
	> 20	3,148	(97.9)	67	(2.1)	2,301	(65.9)	1,014	(29.0)	178	(5.1)
life characteristics	number of household members										
	1	198	(98.5)	3	(1.5)	2,752	(95.9)	94	(3.3)	24	(0.8)
	2	431	(98.2)	8	(1.8)	3,500	(93.4)	211	(5.6)	38	(1.0)
	3–5	6,046	(98.7)	81	(1.3)	15,359	(84.7)	2,377	(13.1)	391	(2.2)
	6–9	677	(99.6)	3	(0.4)	1,394	(81.5)	283	(16.5)	33	(1.9)
	household types										
	single-person household	198	(98.5)	3	(1.5)	2,752	(95.9)	94	(3.3)	24	(0.8)
	couple	339	(97.7)	8	(2.3)	2,802	(93.0)	178	(5.9)	33	(1.1)
	couple + children	5,100	(98.7)	69	(1.3)	9,415	(80.8)	1,911	(16.4)	326	(2.8)
	couple + parents	103	(100.0)	0	NA	1,296	(94.1)	72	(5.2)	9	(0.7)
	couple + children + parents	1,303	(99.2)	11	(0.8)	2,779	(82.3)	530	(15.7)	67	(2.0)
	others	309	(98.7)	4	(1.3)	3,961	(95.0)	180	(4.3)	27	(0.6)
	dormitory										
	yes	7,337	(98.7)	94	(1.3)	22,694	(86.8)	2,954	(11.3)	485	(1.9)
	no	15	(93.8)	1	(6.3)	311	(96.3)	11	(3.4)	1	(0.3)
exposure to hazardous factors	special health checkups										
	not applicable	4,245	(98.6)	61	(1.4)	8,552	(83.2)	1,484	(14.4)	247	(2.4)
	subject of examination	3,107	(98.9)	34	(1.1)	14,453	(89.4)	1,481	(9.2)	239	(1.5)

Table S3. Participation in workplace health promotion (WHP) programs of employees of semiconductor/liquid crystal display (LCD) workplace by risk levels of chronic diseases

Criteria	Classification	Number of workers in each health-risk group	WHP participants, n (% [a])		WHP: Health camp, n (% [a])		WHP: Personal training, n (% [a])		WHP: Musculoskeletal exercises, n (% [a])		WHP: Counseling, n (% [a])	
total		39,073	457	(1.2)	28	(0.1)	23	(0.1)	99	(0.3)	381	(1.0)
risk levels of chronic diseases	healthy workers	22,342	5	(< 0.1)	0	NA	1	(< 0.1)	1	(< 0.1)	3	(< 0.1)
	suspicious cases of chronic diseases	7,865	40	(0.5)	1	(< 0.1)	3	(< 0.1)	12	(0.2)	33	(0.4)
workers at risk of chronic diseases												
	chronic diseases [b]	8,682	407	(4.7)	27	(0.3)	19	(0.2)	86	(1.0)	340	(3.9)
	hypertension	3,389	271	(8.0)	16	(0.5)	10	(0.3)	50	(1.5)	234	(6.9)
	diabetes	528	143	(27.1)	11	(2.1)	3	(0.6)	38	(7.2)	119	(22.5)
	dyslipidemia	6,379	242	(3.8)	16	(0.3)	16	(0.3)	58	(0.9)	196	(3.1)
risk classification in workers at risk of chronic diseases	low-risk group	3,961	31	(0.8)	5	(0.1)	0	NA	12	(0.3)	25	(0.6)
	intermediate-risk group	3,792	108	(2.8)	10	(0.3)	10	(0.3)	10	(0.3)	91	(2.4)
	high-risk group	929	268	(28.8)	12	(1.3)	9	(1.0)	64	(6.9)	224	(24.1)
10-year ASCVD risk score	< 7.5%	7,352	159	(2.2)	16	(0.2)	7	(0.1)	42	(0.6)	123	(1.7)
	≥ 7.5%	95	11	(11.6)	0	NA	0	NA	5	(5.3)	9	(9.5)
Framingham risk score	< 10%	23,005	284	(1.2)	18	(0.1)	14	(0.1)	59	(0.3)	235	(1.0)
	10 to < 20%	2,965	73	(2.5)	8	(0.3)	5	(0.2)	23	(0.8)	57	(1.9)
	≥ 20%	486	29	(6.0)	0	NA	2	(0.4)	4	(0.8)	26	(5.3)

Abbreviation: ASCVD, atherosclerotic cardiovascular disease

[a] Proportion of the participants in WHP activities to the total number of workers in each health-risk classification.

[b] Hypertension, diabetes, or dyslipidemia
